# Age-Related Differences in Motivation of Recreational Runners, Marathoners, and Ultra-Marathoners

**DOI:** 10.3389/fpsyg.2021.738807

**Published:** 2021-11-05

**Authors:** Dagmara Gerasimuk, Ewa Malchrowicz-Mośko, Arkadiusz Stanula, Eduard Bezuglov, Evgenij Achkasov, Andrzej Swinarew, Zbigniew Waśkiewicz

**Affiliations:** ^1^Institute of Sport Sciences, Jerzy Kukuczka Academy of Physical Education, Katowice, Poland; ^2^Faculty of Sport Sciences, Eugeniusz Piasecki Academy of Physical Education, Poznań, Poland; ^3^Department of Sports Medicine and Medical Rehabilitation, Sechenov First Moscow State Medical University (Sechenov University), Moscow, Russia; ^4^Institute of Materials Science, Faculty of Computer Science and Materials Science, University of Silesia, Chorzów, Poland

**Keywords:** running, motivational structure in sport, marathon, ultramarathon, recreational runners

## Abstract

**Aim:** This study was aimed to investigate the influence of age on the motivations of various types of runners, namely, marathoners, ultra-marathoners, and non-starters.

**Methods:** A total of 1,537 runners including 380 women (24.7%) and 1,157 men (75.3%) took part in the diagnostic survey and completed the motivations of marathoners’ scales questionnaire (MOMS). The effect sizes were estimated.

**Results:** The article presents several statistically significant differences in the impact of age on the motivations of runners in different categories and compares the motivations of marathon runners, ultramarathon runners, and non-starters. The results show that young non-starters decide to run for personal goal achievement, and for both marathon and ultramarathon runners, recognition and competition are important. However, for older people in all groups (non-starters, marathoners, and ultramarathoners), personal goal achievement is of the least importance. Among the oldest runners, the most important motives were self-esteem for non-starters and health orientation for marathoners and ultramarathoners.

## Introduction

Although much has been written in the past about running as a social phenomenon and the motivation to run in the psychological dimension, running, mass running events, marathon tourism, and research devoted to it are currently experiencing a renaissance. Research on the motivations of modern runners is important because much research to date has been conducted in a different socio-cultural context. Running motivations have already been analyzed for variables such as gender, nationality, place of residence, or the age of participants of half and full marathons, ultramarathons, or triathlons. Many studies focused on the motivation to run by gender. Researchers usually found differences between male and female motivations (Running, 1995; López-Fernández et al., 2014; Summers et al., 2016; Poczta and Malchrowicz-Mośko, 2018b; Nikolaidis et al., 2019b). Ogles and Masters (2003) presented a typology of marathon runners based on cluster analysis on motivations. Wicker et al. (2012) investigated characteristics of triathlon participants. Nikolaidis et al. studied age-related aspects of marathon participation, while Poczta et al. investigated age-related aspects of half marathon participation (Malchrowicz et al., 2018; Nikolaidis et al., 2019a). Ferrer et al. (2015) claimed that older people are more motivated to run by physical factors than younger ultramarathon participants. Additionally, Saayman et al. found a statistically significant motivational difference in age among triathletes (Myburgh et al., 2014). Knechtle et al. (2013) investigated nationality aspects in motivational structures of ultramarathon runners. Participation motivations of runners have also been assessed according to the type of event, i.e., a traditional versus non-traditional event (Buning and Walker, 2016). Hautbois et al. (2020) investigated the social impact of sporting events and presented a cluster analysis of marathon participants based on perceived benefits. A factor such as place of residence of runners was also examined (e.g., local runners vs. sports tourists) (Shipway and Jones, 2007; Nowak and Chalimoniuk-Nowak, 2015; Besomi et al., 2017; Poczta and Malchrowicz-Mośko, 2018a; Malchrowicz-Mośko, 2019). Researchers who focused on the motivations of runners were also interested in the experience of the athletes as a factor that potentially affected their motivational structure. The aim of the study by Waśkiewicz et al. (2018) was to examine the relationship between motivation characteristics in ultramarathon runners and sports experience expressed as the number of finished ultramarathons. They discovered that ultramarathon runners had different motivations compared to runners of shorter race distances. This was in accordance with a previous study by Doppelmayr and Molkenthin (2004) about finishers in the Marathon des Sables and other runners. Additionally, many years ago, McCutcheon and Yoakum (1983) investigated the personality attributes of ultramarathon runners compared to short distance runners. A comparison among ultramarathon runners, runners of less than 10 miles, and non-runners indicated no difference in personality traits. Hanson et al. (2015) compared half and full marathon runners with ultra-runners and indicated that ultramarathon participants declared less health orientation and fewer weight concerns but a higher life meaning compared to both investigated groups. Malchrowicz-mośko et al. (2020) investigated how years of running experience (less than 1 year, 1–3 years, 3–5 years, and 5–10 years) influence the motivations of amateur marathon athletes, but the results showed that running experience was not a statistically significant factor. Additionally, Stoll et al. (2000) tried to understand how habitual motives change throughout life and Waśkiewicz et al. (2019) compared the motives of successful marathon runners and inexperienced runners. Researchers claimed that successful marathon finishers did not have different motives compared to runners who were intending to compete in their first marathon. There is an increasing number of studies about demanding disciplines such as triathlon (Knechtle et al., 2011). Recent studies indicated the growing interest in extreme sports such as triathlon, which is beginning to be an important research area. Lovett et al. (2018) studied the motivations for participation in triathlon events along with Lamont and Kennelly (2012), Johansen et al. (2021). It should be noted that the Croft et al. (2007), Lovett et al. (2018), and Brown (2019) studies are three known studies on triathletes that used a modified version of the MOMS. However, few studies have been dedicated to motivations of ultramarathon runners. Frick (2011) examined motives of male and female ultramarathon runners, Krouse et al. (2011) evaluated motives of female ultramarathon participants, Malchrowicz-Mośko and Waśkiewicz (2020) and Thuany et al. (2021) analyzed motivation of an ultramarathoner in relation to their family life and marital status or socioeconomic status. They indicated that the most important motivations for running were related to personal goal achievement, health orientation, and self-esteem. The aim of this study was to investigate the influence of age on the motivations in various types of runners, namely, marathoners, ultra-marathoners, and non-starters. The number of older athletes is increasing with the aging of populations across the developed world, so it is important to know what motivates older athletes to run and what are the motivational differences compared to young athletes.

## Materials and Methods

### Participants and Research Tool

A total of 1,537 runners, including 380 women (24.7%) and 1,157 men (75.3%), completed the motivations of marathoners’ scales (MOMS) questionnaire (four of them were excluded from the analysis while their running status was unclearly defined). Participants aged 18–30 years comprised 23.5% of the respondents, while 35.4% were 31–40 years, 30.5% were 41–50 years, and 11.6% were over 50 years of age. The distribution of the surveyed population by sex, age, and the running group was presented in Table 1. The MOMS questionnaire contains 56 items that were distributed across nine scales (Masters et al., 1993). The nine motivations covered the following four main categories: (1) psychological motives including maintaining or enhancing self-esteem, providing a sense of life meaning, and problem solving or coping with negative emotions; (2) social motives including the desire to affiliate with other runners and to receive recognition or approval from others; (3) physical motives for running including general health benefits and weight concern; and (4) achievement-related motives including competition with other runners and personal goal achievement. The Polish translation of the MOMS questionnaire that was adapted and verified for reliability by Dybała (2013) was used. Answers to items on the MOMS questionnaire are on a seven-point Likert-type scale, where 1 means “not a reason” and 7 represents the “most important reason.” The MOMS scale has been adapted in many countries and is sometimes used for different sports (LaChausse, 2006; Ruiz-Juan and Zarauz Sancho, 2011; Heazlewood et al., 2012; Brown et al., 2018).

**TABLE 1:** Distribution of the surveyed population by sex, age, and running group.

Gender	Group	Age [years]			
		<30	31–40	41–50	>50
Men	RR	71	56	43	9
		39,66%	31,28%	24,02%	5,03%
	MA	118	244	201	74
		18,52%	38,30%	31,55%	11,62%
	U	45	120	128	47
		13,24%	35,29%	37,65%	13,82%
	Total	234	420	372	130
		20,24%	36,33%	32,18%	11,25%
Women	RR	49	34	13	2
		50,00%	34,69%	13,27%	2,04%
	MA	67	71	52	10
		33,50%	35,50%	26,00%	5,00%
	U	16	26	32	9
		19,28%	31,33%	38,55%	10,84%
	Total	132	131	97	21
		34,65%	34,38%	25,46%	5,51%
Total		366	551	469	151
		23,81%	35,85%	30,51%	9,82%

*RR- recreational runners, M – marathoners, U – ultra-marathoners.*

### Study Design

All procedures were performed in accordance with Polish law and were evaluated by the Bioethical Committee at the Jerzy Kukuczka Academy of Physical Education in Katowice, which granted official approval for the research (KB/47/17). The diagnostic survey was conducted in accordance with the Declaration of Helsinki. Because online surveys or questionnaires do not require the completion of a separate participant information sheet or consent form, completion of the survey was deemed to constitute informed consent. The questionnaire was distributed to Polish runners through professional running websites and organizers of running events, who directed runners to the online survey. Participants were informed about the significance of the study and were kindly requested to provide information. The survey was anonymous and confidential.

### Statistics

All statistical calculations were performed using SPSS Statistics 24 (IBM Corporation, Armonk, NY, United States). We used the Kruskal–Wallis test, Dunn–Sidak *post hoc* test which is very sensitive for unequal sample sizes, and Mann–Whitney *U* test. Statistical significance was set at α = 0.05. The effect sizes were also estimated.

## Results

The analysis of the results should start with the statement that the population of respondents who responded *via* the questionnaire is similar to the general population of runners in marathons and ultra-marathons. The gender proportions of research are similar to worldwide standards. Subic (2021) performed a statistical analysis that covered 2,348,505 marathon results achieved during the 2009–2019 period and found that 2,149,719 participants had sex given the following: 1,376,441 (64.03%) were men and 773,278 (35.97%) were women. The author was requested data for the Polish population, and it appeared that 7,974 participants were men (7,637) and 1,885 were women (23.63%). It is a growing tendency because, in the years 2003–2013, the percentage of women was equal to approximately 10% with a single race where women represented 23% of starting population (Stempień, 2014). Another research analyzed the population of ultramarathon runners performed by Ronto (2021) who found that there have never been more women in ultrarunning. Twenty-three percent of participants are female, compared to just 14% 23 years ago. There were 19% of Polish women who participated in ultra-marathons. The data includes the results of 5,010,730 million finishers from over 15,000 races and the study looks at data from 1996 to 2018.

### Differences in the Motivations of Marathoners’ Scales Running Motivation Questionnaire Scales Within Different Age Groups in the Recreational Runners (Non-starters in Any Marathon or Ultra-Marathon) Group

Differences in the nine tested scales on the MOMS questionnaire in the four age groups of runners were investigated in the recreational runners’ group, i.e., not participating in marathons or ultramarathons. Because of the significant non-equivalence of the compared groups, a series of non-parametric Kruskal–Wallis analyses were performed. Six statistically significant results were recorded, including the scope of health orientation scales, weight concern, personal goal achievement, competition, recognition, and affiliation. To determine which specific groups differed from each other, *post hoc* analyses were performed using Dunn–Sidak tests. The results are presented in Table 2. For the health orientation scale, two statistically significant differences were noted. There were significantly higher results in the 41–50 years group compared to people under 30 years of age and compared to the 31–40 years group (*p* = 0.001). For the weight concern scale, only one statistically significant difference was noted (*p* = 0.012). The highest motivations were again recorded in the 41–50 years age group, and the lowest motivations results were in the youngest group. For the personal goal achievement scale, there was also only one statistically significant difference, but this time, it was between the youngest and oldest groups (*p* = 0.001). The lowest motivation results were obtained by people over 51 years of age, and the highest motivations were observed in the youngest group. For the scale of competition, two statistically significant differences were noted (*p* = 0.012). The lowest motivations results were again recorded in those over 51 years of age, and this group differed significantly from the youngest group and runners aged 41–50 years. On the recognition scale, one statistically significant difference was noted (*p* = 0.012). The highest motivations scores on this scale were recorded in people 41–50 years of age, and the lowest motivations in people 31–40 years of age. Finally, only one statistically significant difference was noted for the affiliation scale (*p* = 0.005). The highest motivations results were also recorded in the group of people aged 41–50 years, which was statistically significantly lower than the youngest age group. For life meaning, psychological coping, and self-esteem scales, no statistically significant results were obtained.

**TABLE 2:** Comparison of the level of motivation scales of recreational runners in different age groups.

		*N*	*M*	*SD*	*K-W*	*p*	ω^2^
Health orientation	less than 30 years	120	4,42a	1,13	26,44	0,001	0,10
	31–40 years	90	4,53a	1,27			
	41–50 years	56	5,33b	0,80			
	more than 51 years	10	4,78ab	1,47			
Weight concern	less than 30 years	120	4,21a	1,68	10,93	0,012	0,04
	31–40 years	90	4,50ab	1,62			
	41–50 years	56	5,08b	1,48			
	more than 51 years	10	4,73ab	1,78			
Personal goal achievement	less than 30 years	120	5,73a	0,91	16,99	0,001	0,06
	31–40 years	90	5,34a	1,32			
	41–50 years	56	5,35ab	1,08			
	more than 51 years	10	3,92b	1,53			
Competition	less than 30 years	120	3,51a	1,75	10,95	0,012	0,04
	31–40 years	90	3,06ab	1,56			
	41–50 years	56	3,47a	1,72			
	more than 51 years	10	1,95b	1,08			
Recognition	less than 30 years	120	2,83ab	1,44	11,01	0,012	0,04
	31–40 years	90	2,48a	1,35			
	41–50 years	56	3,23b	1,70			
	more than 51 years	10	1,88ab	0,82			
Affiliation	less than 30 years	120	2,95a	1,43	12,85	0,005	0,05
	31–40 years	90	3,29ab	1,70			
	41–50 years	56	3,83b	1,67			
	more than 51 years	10	2,48ab	1,67			
Psychological coping	less than 30 years	120	4,42	1,59	7,26	0,064	0,03
	31–40 years	90	4,39	1,64			
	41–50 years	56	4,34	1,49			
	more than 51 years	10	2,98	1,29			
Life meaning	less than 30 years	120	4,04	1,42	5,88	0,117	0,02
	31–40 years	90	3,92	1,58			
	41–50 years	56	4,11	1,42			
	more than 51 years	10	2,90	1,32			
Self esteem	less than 30 years	120	4,89	1,34	5,67	0,129	0,02
	31–40 years	90	4,48	1,55			
	41–50 years	56	4,68	1,42			
	more than 51 years	10	4,00	1,30			

*Different letter indexes mean groups that differ from each other at p < 0.05 Dunn-Sidak test.*

*N – number of respondents; M – median; SD – standard deviation; K-W – result of Kruskal-Wallis test; p – statistical significance; ω^2^-effect size.*

### Differences in the Motivations of Marathoners’ Scales Running Motivation Questionnaire Scales Within Different Age Groups in the Marathon Runners’ Group

The score for the nine tested scales was investigated in the four age groups of marathon runners, i.e., those participating in marathons and not in ultramarathons. Up to six statistically significant results were recorded in the scope of weight concern scales, personal goal achievement, recognition, psychological coping, life meaning, and self-esteem. The results are presented in Table 3. For the weight concern scale, two statistically significant differences were noted (*p* = 0.011). The lowest motivations results were recorded in the oldest age group, i.e., runners over 51 years old. This group differed from the youngest and those aged 31–40 years. Other pairs of groups did not differ statistically. For the personal goal achievement scale, the lowest motivations results were again recorded in the oldest group. The differences between this group and the three other groups were statistically significant (*p* = 0.001). In addition, a statistically significant difference was noted between the youngest group that had the highest motivations scores on the studied scale and the 41–50 years group. Other pairs of groups did not differ. For the recognition scale, two statistically significant differences were noted (*p* = 0.007), and the highest motivations scores on this scale were recorded in the youngest group, while the scores were significantly lower in the 31–40 years and in the over 51 years age groups. For the psychological coping scale, two statistically significant differences were noted (*p* = 0.001). The highest motivations results were recorded in the group below 30 years of age, while significantly lower scores were reported in the two oldest age groups. For the other two scales, life meaning (*p* = 0.002) and self-esteem (*p* = 0.001), three statistically significant differences were noted. Both scales recorded the highest motivation results in the group that was below 30 years of age, and this group differed significantly from the other three age groups, which, in turn, did not differ from each other even at the level of statistical tendency. For health orientation and affiliation scales, the Kruskal–Wallis test results were not significant or even close to statistical significance.

**TABLE 3:** Comparison of the level of motivation scales of marathon runners not competing in ultramarathons in different age groups.

		*N*	*M*	*SD*	*K-W*	*p*	ω^2^
Health orientation	less than 30 years	184	4,53	1,12	5,24	0,155	0,01
	31–40 years	309	4,67	1,17			
	41–50 years	254	4,72	1,09			
	more than 51 years	85	4,77	1,21			
Weight concern	less than 30 years	184	4,81a	1,61	11,21	0,011	0,01
	31–40 years	309	4,78a	1,65			
	41–50 years	254	4,65ab	1,61			
	more than 51 years	85	4,13b	1,70			
Personal goal achievement	less than 30 years	184	5,72a	0,96	37,36	0,001	0,04
	31–40 years	309	5,46ab	1,09			
	41–50 years	254	5,29b	1,18			
	more than 51 years	85	4,74c	1,39			
Competition	less than 30 years	184	3,63T	1,75	8,84	0,032	0,01
	31–40 years	309	3,38	1,57			
	41–50 years	254	3,23	1,63			
	more than 51 years	85	3,08T	1,69			
Recognition	less than 30 years	184	3,03a	1,59	12,26	0,007	0,01
	31–40 years	309	2,59b	1,30			
	41–50 years	254	2,74ab	1,45			
	more than 51 years	85	2,45b	1,45			
Affiliation	less than 30 years	184	3,26	1,58	5,74	0,125	0,01
	31–40 years	309	3,28	1,59			
	41–50 years	254	3,54	1,66			
	more than 51 years	85	3,58	1,72			
Psychological coping	less than 30 years	184	4,74a	1,47	19,08	0,001	0,02
	31–40 years	309	4,49abT	1,41			
	41–50 years	254	4,27b	1,47			
	more than 51 years	85	4,02bT	1,39			
Life meaning	less than 30 years	184	4,40a	1,42	14,54	0,002	0,02
	31–40 years	309	4,01b	1,40			
	41–50 years	254	3,88b	1,44			
	more than 51 years	85	3,87b	1,42			
Self esteem	less than 30 years	184	5,25a	1,20	43,56	0,001	0,05
	31–40 years	309	4,56b	1,33			
	41–50 years	254	4,52b	1,34			
	more than 51 years	85	4,34b	1,47			

*Different letter indexes mean groups differing at the level of p < 0.05 Dunn-Sidak test.*

*N – number of respondents; M – median; SD – standard deviation; K-W – the result of Kruskal-Wallis test; p – statistical significance; ω^2^- effect size.*

### Differences in the Motivations of Marathoners’ Scales Running Motivation Questionnaire Scales Within Different Age Groups in the Ultramarathon Runners’ Group

We then investigated whether the scores for the nine scales were different in the four age groups for ultramarathon runners. The results are presented in Table 4. For the personal goal achievement scale (*p* = 0.001), the lowest motivations results were found in the oldest group. The differences between this group and the youngest and the 31–40 years age groups were statistically significant. For the scale of competition, only one statistically significant difference was noted (*p* = 0.014). People under the age of 30 years had a significantly higher level of this scale than people over 51 years of age. For the recognition scale, two statistically significant differences were noted (*p* = 0.004). The highest motivations results were recorded for the youngest runners. This group was statistically significantly different from the 41–50 years and over 51 years of age groups.

**TABLE 4:** Comparison of the level of motivation scales of ultramarathon runners not competing in marathons in different age groups.

		*N*	*M*	*SD*	*K-W*	*p*	ω^2^
Health orientation	less than 30 years	62	4,27	1,21	5,14	0,162	0,01
	31–40 years	146	4,57	1,08			
	41–50 years	160	4,59	1,17			
	more than 51 years	57	4,67	1,17			
Weight concern	less than 30 years	62	4,29	1,51	1,24	0,743	0
	31–40 years	146	4,32	1,69			
	41–50 years	160	4,41	1,73			
	more than 51 years	57	4,14	1,72			
Personal goal achievement	less than 30 years	62	5,49a T	1,04	18,79	0,001	0,04
	31–40 years	146	5,23a	1,22			
	41–50 years	160	5,01ab T	1,23			
	more than 51 years	57	4,52b	1,39			
Competition	less than 30 years	62	3,83a	1,53	10,55	0,014	0,02
	31–40 years	146	3,30ab	1,63			
	41–50 years	160	3,38ab	1,65			
	more than 51 years	57	2,94b	1,65			
Recognition	less than 30 years	62	3,11a	1,43	13,55	0,004	0,03
	31–40 years	146	2,73ab	1,46			
	41–50 years	160	2,52b	1,31			
	more than 51 years	57	2,24b	1,20			
Affiliation	less than 30 years	62	3,30	1,62	6,14	0,105	0,01
	31–40 years	146	3,77	1,54			
	41–50 years	160	3,41	1,57			
	more than 51 years	57	3,71	1,74			
Psychological coping	less than 30 years	62	4,36	1,47	6,67	0,083	0,02
	31–40 years	146	4,49	1,32			
	41–50 years	160	4,15	1,54			
	more than 51 years	57	3,90	1,55			
Life meaning	less than 30 years	62	4,34	1,46	1,49	0,685	0
	31–40 years	146	4,26	1,32			
	41–50 years	160	4,12	1,40			
	more than 51 years	57	4,15	1,53			
Self esteem	less than 30 years	62	4,77	1,34	10,17	0,017	0,02
	31–40 years	146	4,59	1,25			
	41–50 years	160	4,30	1,37			
	more than 51 years	57	4,10	1,55			

*Different letter indexes mean groups differing at the level of p < 0.05 Dunn-Sidak test.*

*N – number of respondents; M – median; SD – standard deviation; K-W – the result of Kruskal-Wallis test; p – statistical significance; ω^2^ - effect size.*

## Discussion

During the last decades, the popularity of mass running events has been described as a “marathon fever” (Summers et al., 1982). Today, we also observe a similar tendency among ultramarathon runners. Many new runners of different ages also want to start the adventure with running. Based on our results, it is possible to compare the most and least important motivations for the youngest and the oldest runners (Table 5).

**TABLE 5:** The youngest runners vs. the oldest runners (statistically significant differences).

	The youngest runners below 30	The oldest runners over 51
**NON-STARTERS**		
The most important aspects	Personal goal achievement	
The least important aspects		Personal goal achievement
		Affiliation
		Competition
**MARATHONERS**		
The most important aspects	Recognition	
	Psychological coping	
	Life-meaning	
	Self-esteem	
The least important aspects		Weight concern
		Personal goal achievement
**ULTRAMARATHONERS**		
The most important aspects	Competition	
	Recognition	
	Self-esteem	
The least important aspects		Personal goal achievement

The number of older athletes is increasing with the aging of populations across the developed world, therefore, it is important to know what motivates older athletes to run (Dionigi, 2006). Contemporary aging adults are not content only with Nordic walking, swimming, or dancing, but they are choosing increasingly demanding disciplines in terms of endurance and technology, such as surfing or participation in a marathon (Wheaton, 2017).

Some of the research results on runner age and motivation are consistent with the few previous studies in this area. According to Ogles and Masters (2000) who investigated only male athletes, older runners were more motivated by a general health orientation and young runners were more motivated by personal goal achievement. Nikolaidis et al. (2019a) also indicated that younger male athletes scored higher for competition aspects. The most important difference that Poczta et al. found in their analysis between younger and older half marathon participants was that younger runners were more focused on sports results while older runners were more often focused on social aspects and contact with other half-marathoners (Malchrowicz et al., 2018). They found that for older respondents who participated in events that were more demanding than a half-marathon, affiliation was not among the dominant factors of participation, but it showed an upward trend depending on the increasing difficulty of the run: from 3.58 among marathoners to 3.71 among ultramarathoners. High results for affiliation in older ultramarathoners is consistent with a qualitative study by Kazimierczak et al. (2020), who highlighted the importance of affiliation and social identity for ultramarathoners. For older respondents, health orientation was especially important. However, health control and educational information before races might be a necessary step to avoid life-threatening complications because studies have shown that runners are often unable to stop despite disturbing symptoms and the desire to finish the run may expose them to life-threatening effects (Khorram-Manesh et al., 2019).

## Conclusion

Our work extends the current knowledge about motivation in sport. It shows how age influences the motivations of different types of runners. These research results show that young recreational runners decide to run to achieve a personal goal, and for both marathon and ultramarathon runners, recognition and competition are important. For older people in all groups personal goal achievement is of the least importance, while among the oldest runners, the most important motives were self-esteem for non-starters and health orientation for marathoners and ultramarathoners. The least important motive for all categories of old runners was recognition.

## Study Limitation and Future Directions

The key strength of this study is the large sample size while limitations involved the use of an online study to obtain the data. However, online studies were reported to obtain very similar results compared to those that are administered manually using paper and pencil (Van De Looij-Jansen and De Wilde, 2008; Hohwü et al., 2013). In the future, more characteristics of runners should be investigated, e.g., marital status as it is a potentially important factor in the field of motivation to run (Lev and Zach, 2018). Age-related motives by gender should also be examined. Lastly, sport is an area in which masculinity and youth along with their achievements are celebrated and rewarded. Thus, aging women face a double barrier when they wish to participate in a competitive sport (Pfister, 2012).

## Data Availability Statement

The raw data supporting the conclusions of this article will be made available by the authors, without undue reservation.

## Ethics Statement

The studies involving human participants were reviewed and approved by Jerzy Kukuczka Academy of Physical Education. The patients/participants provided their written informed consent to participate in this study.

## Author Contributions

ZW and DG: conceptualization, methodology, and investigation. ZW, AnS, and ArS: software, validation, and data curation. ZW and EM-M: formal analysis and resources. ZW, EM-M, and EB: writing—original draft preparation. EM-M, ZW, AnS, ArS, EB, and DG: writing—review and editing. EM-M: visualization. ZW: supervision and project administration. All authors have read and agreed to the published version of the manuscript.

## Conflict of Interest

The authors declare that the research was conducted in the absence of any commercial or financial relationships that could be construed as a potential conflict of interest.

## Publisher’s Note

All claims expressed in this article are solely those of the authors and do not necessarily represent those of their affiliated organizations, or those of the publisher, the editors and the reviewers. Any product that may be evaluated in this article, or claim that may be made by its manufacturer, is not guaranteed or endorsed by the publisher.
